# Isolation, detection of virulence genes, antibiotic resistance genes, plasmid profile, and molecular typing among *Vibrio parahaemolyticus* isolated in Malaysian seawater from recreational beaches and fish

**DOI:** 10.14202/vetworld.2019.1140-1149

**Published:** 2019-07-28

**Authors:** Orooba Meteab Faja, Ali Abd Sharad, Khansa Mohammed Younis, Merriam Ghadhanfar Alwan, Basima Jasim Mohammed, Asmat Ahmad

**Affiliations:** 1 Department of Public Health, College of Veterinary Medicine, University of Al-Qadisiyah, Iraq; 2 Department of Biology, College of Education for Pure Science, Anbar University, Iraq; 3 Department of Biology and Microbiology, Faculty of Sciences, Mosul University, Mosul, Iraq; 4 Department of Biology, School of Bioscience and Biotechnology, Faculty of Science and Technology, The National University of Malaysia 43600 UKM, Bangi, Selangor, Malaysia

**Keywords:** antibiotic resistance genes, plasmid profile, *Vibrio parahaemolyticus*, virulence genes

## Abstract

**Background and Aim:**

Despite the importance of the global emergence of *Vibrio* *parahaemolyticus* infections worldwide, there has been scanty information on its occurrence in Malaysian seawaters and fish. This study aimed to determine the occurrence of *V. parahaemolyticus* isolates using polymerase chain reaction targeted at toxin operon gene, thermostable direct hemolysin (*tdh*), and *tdh*-related hemolysin genes and to determine antibiotic resistance pattern, genes, and plasmid profile of *V. parahaemolyticus* from Malaysian seawaters and fish.

**Materials and Methods:**

Samples were collected from four recreational beaches in Malaysia (Port Klang; Bachok; Port Dickson; and Mersing). Thiosulfate-citrate-bile salts-sucrose (TCBS) agar and chromogenic *Vibrio* agar were used for isolation and identification. Colonies with yellow color on TCBS and green color on chromogenic vibrio (CV) agar were considered to be *V. parahaemolyticus* and they were subjected to biochemical tests. All *V. parahaemolyticus* isolates were further subjected to identification using seven specific gene markers.

**Results:**

Seventy-three *Vibrio* isolates were recovered. Only one gene thermostable direct hemolysin (*tdh*) from seawater isolates of *Vibrio* has high virulence gene percentage (95.23%). Two genes alkaline serine protease (*asp*) and (*tdh*) had high percentage of virulence (83.87% and 80.64%, respectively) from fish. Comparatively, fish isolates have a higher virulence percentage compared to seawater isolates. Only gene streptomycin resistance B (*strB*) from seawater had 100% of the resistance genes. All isolates were multi-antibiotic resistant. Seventeen antibiotic resistance patterns were observed. The isolates had plasmids of varying sizes ranging from 2.7 kb to 42.4 kb. Dendrogram based on antibiotic resistance patterns of *V. parahaemolyticus* isolates discriminated the isolates into three clusters.

**Conclusion:**

This study demonstrated the occurrence of pathogenic, multi-antibiotic-resistant *V. parahaemolyticus* strains in Malaysian coastal waters and fish, and this could constitute potential public health risks.

## Introduction

The genus *Vibrio* consists of aquatic microbes which normally live in coastal and estuarine water bodies [1]. The bacteria are known to be ubiquitous in these environments as they have been isolated from seawater, fish, and shellfish [2]. One of the pathogenic species of *Vibrio* that is commonly isolated from coastal and estuarine water bodies all over the world is *Vibrio* *parahaemolyticus*. It is a Gram-negative halophilic bacterial [3] and it is present in abundance. The distribution of *V*. *parahaemolyticus* in the marine and estuarine environments has been reported to vary based on the temperature of the water [2,4]. The consumption of foods contaminated with a high level of *V. parahaemolyticus* and/or pathogenic *V. parahaemolyticus* has been associated with gastrointestinal infections [5,6]. Concomitantly, *V. parahaemolyticus* infections in humans have been frequently reported in coastal areas as a result of increased consumption of seafood and direct contact with *Vibrio* contaminated estuarine waters [7]. Among *Vibrio* species of clinical importance, *V. parahaemolyticus* produces virulence factors that encode the thermostable direct hemolysin (*tdh*) and/or the *tdh*-related hemolysin (*trh*); which have been reported to contribute to the pathogenicity of the species [8]. Resistance to antimicrobials, on the other hand, has now been acknowledged as a critical threat to public health as well as food security globally [9]. Most of the common antibiotics that are frequently utilized are no longer effective. The widespread use and misuse of antibiotics in aquaculture, agriculture, and livestock production have been considered to be one of the fundamental factors influencing the emergence and spread of antimicrobial resistance. Multidrug-resistant bacterial strain is another emerging challenge when a bacterial cell becomes resistant toward multiple antibiotics [10].

Despite the importance of the global emergence of *V. parahaemolyticus* infections worldwide in the coastal areas, there has been scant information on the diversity of *V. parahaemolyticus* in Malaysian coastal waters and fish. This information is readily available, could be used for better policy formulation and implementation, thereby mitigating the increasing rate of human *V. parahaemolyticus* infection visa-vise antibiotic resistance.

This study aimed to determine the presence of *V. parahaemolyticus* isolates to the species level using polymerase chain reaction (PCR) targeting the toxin operon (*toxR*) gene, to determine the virulence factors using PCR technique directed at *tdh* and *trh* genes and to determine antibiotic resistance and plasmid profile of *V. parahaemolyticus* isolates from Malaysian coastal waters and fish.

## Materials and Methods

### Ethical approval

Samples were collected as per standard sample collection method. There is no need to obtain ethical approval for such type of study.

### Bacterial isolation and identification

The isolation of *V. parahaemolyticus* from seawater and fish (Sea bass) was done using Sterile 500 mL Schott bottles for the collection of seawater [11], while sterile disposable plastic containers were used for fish collection [12]. The samples were collected from four recreational beaches of Malaysia (Port Klang, Selangor; Bachok, Kelantan; Port Dickson, Negeri Sembilan; and Mersing, Johor). Serial dilution method and spread plate technique were employed for culturing *V. parahaemolyticus*. Thiosulfate-citrate-bile salts-sucrose (TCBS) Agar-TCBS (Oxoid, UK) and Chromogenic *Vibrio* Agar CV (Titan Media, India) were used for isolation and identification of bacteria. The colonies that appeared yellow on TCBS agar and green on CV agar were considered as *V. parahaemolyticus* and they were subjected to biochemical tests following Food and Drug Administration (FDA) and Bergey’s manuals [13].

### DNA and plasmid extraction

For the DNA extractions, DNA was extracted from the 73 *V. parahaemolyticus* isolates using DNA Purification Kit (Promega, USA), following the manufacturer’s manual. The extracted genomic DNA was then stored at −20°C for further studies. For plasmid extractions, *V. parahaemolyticus* was cultivated overnight in 5 ml of Luria Bertani broth (Oxoid, UK) with an addition of 4% (w/v) NaC1 at 35°C, using FavorPrep™ Plasmid Extraction Mini Kit (Favorgen Biotech Corp., Taiwan) following manufacturer’s instruction. The plasmid DNA products were stored at −20°C for further analysis. The final products of both processes were then loaded in 1% (w/v) agarose gel. The gel electrophoresis was run at 85 V for 1 h. One kb Extend DNA ladder (New England Biolabs, USA) and 1 kb DNA ladders (Invitrogen, Belgium) were used as DNA markers. The gel was then visualized and recorded under ultraviolet (UV) light using GeneSys G: BOX EF2 (Syngene, USA) device [14].

### Molecular identification of *V. parahaemolyticus*

All *V. parahaemolyticus* isolates that were identified biochemically were then subjected to identification using specific gene markers. Briefly, DNA gyrase subunit B (*gyr*B) gene with primer sequences of *gyr*B-F 5’-CGG CGT GGG TGT TTC GGT AGT-3’) (*gyr*B –R 5’-TCC GCT TCG CGC TCA TCA ATA-3’ as described previously [15] was used to identify *V. parahaemolyticus*. Identification by 16s rRNA gene sequencing was equally done using the following primes (27F 5’-AGA GTT TGA TCM TGG CTC AG-3’) and (1492R 5’ –TAC GGY TAC CTT GTT ACG ACT T -3’) as previously described [16] and some of the isolates were submitted to GenBank with accession numbers as shown in Table-1.

**Table 1:** The sequences similarity of the 16S rRNA gene of *V. parahaemolyticus* strains isolated from Malaysia with those previously deposited in NCBI (GenBank) database from other related studies.

Isolate code	GenBank accession number	Isolate names	Identification (% identity)
VPCW3	MF278586.1	*V.parahaemolyticus* strain ukmVp1	91
VPDW1	MH071288.1	*V.parahaemolyticus* strain ukmVp2	99
VPDS4	MF347992.1	*V.parahaemolyticus* strain ukmVp3	93
VPIS1	MH021958.1	*V.parahaemolyticus* strain ukmVp4	99
VPKS3	MH071289.1	*V.parahaemolyticus* strain ukmVp5	97
VPLW1	MH071290.1	*V.parahaemolyticus* strain ukmVp6	95

*V.* *parahaemolyticus=Vibrio* *parahaemolyticus*

### Detection of virulence genes

A total of 73 isolates (42 recovered from seawater and 31 from fish) of *V. parahaemolyticus* were screened for the presence of virulence genes. PCR assays were applied to target the virulence determinant *tdh* gene, *trh* gene, thermolabile hemolysin gene (*tlh*), *tox*R gene, outer membrane protein, and alkaline serine protease (*asp*) [18,19]. Details of primers used are listed in Table-2. For each primer, initial optimization experiments were conducted to ascertain optimal PCR conditions for MgCl_2_ and annealing temperatures and the reaction mixture of the total volume of 25 μL was prepared. PCR conditions used are presented in Table-3. Reference strains (*V. parahaemolyticus* ATCC 17802, *V. parahaemolyticus* ATCC 43996, and *Escherichia coli* ATCC 25992) were used to standardized the reaction. All reactions were conducted in a Mastercycler^®^ thermal cycler (Eppendorf, Germany). Gel electrophoresis assay was done by mixing 10 μL of the amplicon with 2 μL of gel loading dye and electrophoresed in pre-stained 1.0% agarose (Sigma-Aldrich, USA) for 1 h at 80-90 V in 1× Tris-Acetate Ethylenediaminetetraacetic acid (TAE) buffer (FirstBase Sdn Bhd, Malaysia). The 100 bp and 1 kb DNA ladders (Invitrogen, Belgium) were used as a molecular size marker.

**Table 2:** Primer used to detect virulence genes in this study.

Gene name	Primer name	Primer sequence (5’ to 3’)
*tdh*	*tdh*-F	CCATTCTGGCAAAGTTATT
	*tdh*-R	TTCATATGCTTCTACATTAAC
*trh*	*trh*-F	TTGGCTTCGATATTTTCAGTATCT
	*trh*-R	CATAACAAACATATGCCCATTTCCG
*tlh*	*tlh*-F	AGCGGATTATGCAGAAGCAC
	*tlh*-R	ATCTCAAGCACTTTCGCACG
*toxR*	*toxR*-F	GATTAGGAAGCAACGAAAG
	*toxR*-R	GCAATCACTTCCACTGGTAAC
*ompK*	*ompK*-F	GGCGGTCGCTCTGGTATT
	*ompK*-R	TTGCCATCGTAAGTGCTGTA
*asp*	*asp*-F	CGAAGCGGGCTGGGGTTA
	*asp*-R	ACATGCGGTGTGGCCATAGAGG
*colA*	*colA*-F	CGAGTACAGTCACTTGAAAGCC
	*colA*-R	CACAACAGAACTCGCGTTACC

*tdh*=Thermostable direct hemolysin, *trh=*TDH-related hemolysin, *tlh*=Thermolabile hemolysin, *toxR*=Toxin operon, *ompK:* Outer membrane protein, *asp=*Alkaline serine protease, *colA=*Collagenase

**Table 3:** Antibiotic resistance patterns and plasmid profiling of *Vibrio* *parahaemolyticus*.

Antibiotics	Isolate codes	Pattern	Plasmid Size (kb)
AM, AX, C, CN, E, F, K, KF, NA, P, PY, S, TE	VPDW1, VPKW1, VPLW1	A	3.8,7.3, 10.2, 15, 42.4
AM, AX, C, CIP, E, F, K, KF, P, PY, S, TE	VPKW2, VPLW2	B	3.8, 7.3, 13.5, 42.4
AM, AX, C, E, F, K, NA, P, PY, S, TE	VPCW3. VPEW2, VPFW4, VPHW4, VPIW1, VPJW3	C	2.7, 3.8, 10.2
AM, AX, E, F, K, P, PY, S, TE	VPLW3	D	5.6, 8.2
AM, AX, CN, P, PY, S, TE	VPAW1, VPBW2, VPCW1, VPCW8, VPEW1, VPGW1, VPHW1, VPHW2, VPIW4, VPIW9, VPJW5, VPKW3, VPKW5	E	2.7, 3.8, 42.4
AM, AX, F, P, PY, S, TE	VPEW3, VPKW4	F	32
AM, NA, P, PY, S	VPCW4, VPGW5	G	ND
AM, PY, S	VPAW4, VPBW4, VPCW2, VPCW6, VPCW7, VPHW10, VPHW3, VPHW6, VPIW6, VPJW1, VPJW2, VPJW4, VPJW6	H	ND
AM, AX, C, CIP, CN, E, F, K, NA, P, PY, S, TE	VPDS4, VPIS1, VPKS3	I	2.7, 13.5, 15, 32
AM, AX, C, E, KF, NA, P, PY, S, TE	VPKS1, VPLS2	J	3.8, 5.6, 8.2
AM, AX, CN, E, K, NA, P, PY	VPCS2, VPES1	K	ND
AM, AX, K, P, PY, S, TE	VPKS5, VPLS3	L	10.2, 42.4
AM, CN, K, KF, S, TE	VPAS4, VPBS1, VPCS1, VPDS1, VPKS6, VPLS4	M	6, 13.5
AM, AX, K, P, PY, S	VPAS1, VPBS2, VPDS2, VPLS1	N	ND
AM, AX, NA, P, PY	VPBS3, VPDS5, VPHS3, VPJS1	O	ND
AM, AX, P, PY	VPAS5, VPCS3, VPHS1, VPJS3	P	ND
AM, P, PY	VPAS3, VPAS6, VPCS4, VPIS2	Q	ND

ND=Not detected, AM=Ampicillin, AX=Amoxicillin, C=Chloramphenicol, CN=Gentamycin, E=Erythromycin, F=Nitrofurantoin, K=Kanamycin, KF=Cephalothin, NA=Nalidixic acid, P=Penicillin, PY=Carbenicillin, S=Streptomycin, TE=Tetracycline, CIP=Ciprofloxacin, E=Erythromycin, F=Nitrofurantoin, K=Kanamycin

### PCR amplification of antibiotic resistance genes

All *V. parahaemolyticus* isolates (n=73) were screened phenotypically for the presence of antibiotic resistance genes using 14 different antibiotics (ampicillin [10 μg], amoxicillin [10 μg], chloramphenicol [30 μg], ciprofloxacin [10 μg], gentamycin [10 μg], erythromycin [15 μg], nitrofurantoin [200 μg], kanamycin [30 μg], cephalothin [30 μg], nalidixic acid [30 μg], penicillin [10 µg], carbenicillin [100 μg], streptomycin [25 μg], and tetracycline [30 μg]) (Oxoid, UK) through disc diffusion method [20] and followed by genotype screening using PCR technique to detect the carriage of genes coding for resistance to antibiotics. The following resistant genes; streptomycin resistance (*strB*), β-lactamase resistance (*blaP1*), chloramphenicol resistance (*floR*), tetracycline resistance (*tetA*), erythromycin resistance (*ermB*), quinolone resistance protein (*qnrA*), and aminoglycosides resistance (*aac*(3)-*IIa*) were employed in this investigation for detection of genes responsible for resistance as previously described [21,22]. The primers’ list and PCR reaction conditions are listed in Tables-2 and 4, respectively [18,19]. All reactions were conducted in a Mastercycler^®^ thermal cycler (Eppendorf, Germany). Positive and negative controls were included for each set of amplification. A 100 bp and 1 kb DNA ladder (Invitrogen, Belgium) were used as molecular size markers.

**Table 4:** Polymerase chain reaction conditions of detection of virulence genes.

Gene	Size (bp)	Primer Conc.	dnTP (μM)	*Taq* poly. (units)	MgCl_2_ (mM)	Buffer	Anneal Temp (^o^C)	References
*tdh*	534	0.5	200	0.04	2.0	1x	48	[17]
*trh*	500	0.5	200	0.04	2.0	1x	52	[17]
*tlh*	150	0.5	200	0.04	2.0	1x	54	[17]
*toxR*	658	0.5	200	0.04	2.0	1x	54	[17]
*ompK*	319	0.5	200	0.04	2.0	1x	57	[18]
*asp*	750	0.5	200	0.04	2.0	1x	53	[18]
*colA*	737	0.5	200	0.04	2.0	1x	58	[19]

*tdh*=Thermostable direct hemolysin, *trh=*TDH-related hemolysin, *tlh*=Thermolabile hemolysin, *toxR*=Toxin operon, *ompK*=Outer membrane protein, *asp=*Alkaline serine protease, *colA=*Collagenase

### Molecular typing of *V. parahaemolyticus* by randomly amplified polymorphic DNA (RAPD)-PCR

To assess the utility of RAPD-PCR in the subtyping of *V. parahaemolyticus* recovered during the study, all isolates were subjected to RAPD-PCR. The Gen1 5’-AGGATACGTG-3’ primer [23] was used for the RAPD-PCR reactions. *Vibrio vulnificus* ATCC 27562 was used as a reference and negative controls were performed by adding 1 μL of sterile ultrapure deionized water [24]. PCR amplifications were performed in 0.2 mL and the total volume of the reaction mixture was 50 µL consisting of 25 µL 10× PCR master mix (EconoTaq^®^ Plus Green 2X Master Mix, Lucigen, UK), 0.5 µL of primer and template DNA 1.0 µL and then the volume adjusted to the final volume by addition of nuclease-free water (NFW). PCR conditions adopted involving initial denaturation at 95°C for 5 min followed by 45 cycles of denaturation at 94°C for 1 min, annealing for 1 min at 35°C, and polymerization at 72°C for 2 min. Final elongation was at 72°C for 7 min [25].

### Molecular typing of *V. parahaemolyticus* by enterobacterial repetitive intergenic consensus (ERIC)-PCR

The ERIC-PCR assay was performed on all *V. parahaemolyticus* isolates that were recovered during the study. The PCR technique was carried out in 0.2 ml microcentrifuge tubes, with a 50 µL reaction mixture consisting of 25 µL of 2X DreamTaq Green PCR Master Mix (Thermo Scientific, USA), 1 µL of 100 µM of each ERIC primers as described previously by Versalovic *et al*. [24]; ERIC-1 (5’- CAC TTA GGG GTC CTC GAA TGT A -3’) and ERIC-2 (5’- AAG TAA GTG ACT GGG GTG AGC G -3’) [25], 1 µL of approximately 100 ng DNA template. This was followed by adjusting the volume to 50 µL by adding NFW. Both positive and negative, DNA controls were included in each reaction. The cycling conditions were as follows: Pre-denaturation at 95°C for 7 min, denaturation at 90°C for 30 s, annealing at 58°C for 1 min, and extension at 65°C for 8 min, and with a final extension at 68°C for 16 min at the end of 30 cycles.

### RAPD and ERIC-PCR analysis

A 10 µL volume of each PCR product was mixed with 2 μL of loading dye and subjected to a 1% TAE buffer electrophoresis system. In each run, a molecular weight marker 100 bp PCR ladder (Invitrogen) was included. Following the completion of electrophoresis, the gel was photographed using a UV transilluminator and the relatedness of the bacterial isolates was estimated by ERIC-PCR and RAPD-PCR according to the photographic image of the gel using ImageJ. The data obtained then were clustered using average linkage (unweighted group pair method with arithmetic averages, [UPGMA]) using PHYLIP (version 3.697) (University of Washington, USA) application and the analysis was performed in dendrogram form.

## Results

### Percentage of virulence genes of *V. parahaemolyticus*

**Figure-1:**
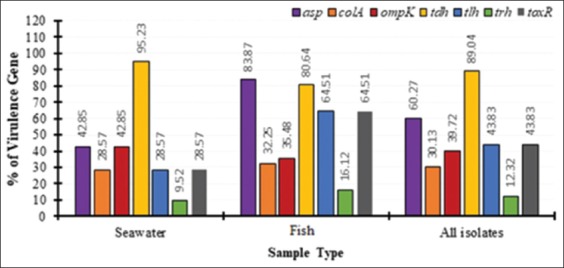
Percentage of virulence genes of Vibrio parahaemolyticus from seawater isolates, isolates from fish and all isolates.

The percentages of virulence genes of *V. parahaemolyticus* from seawater, fish, and all isolates that were recovered during this study are depicted in Figure-1. Seven genes were evaluated, and our analysis revealed that only one gene from seawater isolates has a high virulence gene percentage, and this was gene *tdh*, which had 95.23% virulence (Table-5). The gene with the least virulence percentage from seawater isolate was found to be gene *trh* (9.52%). Genes from isolates of fish, on the other hand, revealed that two of the genes have a high percentage of virulence and these were *asp* and *tdh* genes which had 83.87% and 80.64% of virulence, respectively, while two other genes (*tlh* and *tox*R) had 64.51% virulence each. The gene with the least virulence percentage from the fish isolate was *trh*, which had 16.12% virulence. However, only one gene from all isolate had a high percentage of virulence and this was *tdh* with the virulence of 89.04%, and one other gene (*asp*) had 60.27% of virulence. The gene with the least virulence percent from all isolates was *trh* gene with the virulence of 12.32%. Comparatively, isolates from fish had genes with higher virulence percentage compared to seawater isolates.

**Table 5:** Percentage of virulence genes of *Vibrio* *parahaemolyticus.*

Gene	Seawater n=42 (%)	Fish n=31 (%)	All isolates n=73 (%)
*asp*	18 (42.85)	26 (83.87)	44 (60.27)
*colA*	12 (28.57)	10 (32.25)	22 (30.13)
*ompK*	18 (42.85)	11 (35.48)	29 (39.72)
*tdh*	40 (95.23)	25 (80.64)	65 (89.04)
*tlh*	12 (28.57)	20 (64.51)	32 (43.83)
*trh*	4 (9.52)	5 (16.12)	9 (12.32)
*toxR*	12 (28.57)	20 (64.51)	32 (43.83)

*asp=*Alkaline serine protease, *colA=*Collagenase, *ompK=*Outer membrane protein, *tdh*=Thermostable direct hemolysin gene, *tlh*=Thermolabile hemolysin, *trh=*TDH-related hemolysin, *toxR*=Toxin operon

### Antibiotic resistance patterns and plasmid profile of *V. parahaemolyticus*

**Figure-2:**
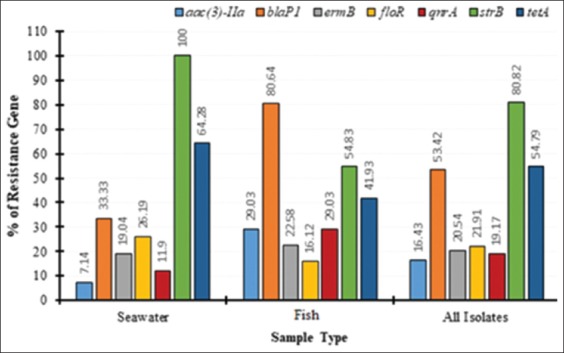
Percentages of seven resistance genes of *Vibrio parahaemolyticus* from seawater isolates, isolates from fish and all isolates.

**Figure-3:**
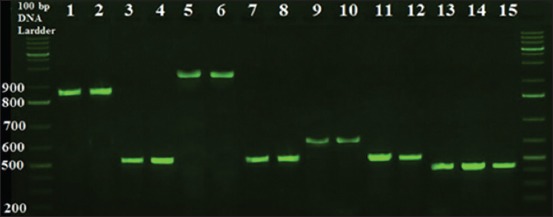
Detection of antibiotic resistance genes in *Vibrio parahaemolyticus* isolates by polymerase chain reaction technique, electrophoresed on 1.5% (w/v) agarose gel. Lanes 1, 2: β-lactamase resistance gene in VPEW3 and VPGW5. Lanes 3, 4: streptomycin resistance gene in VPAW1 and VPBW2. Lanes 5, 6: tetracycline Resistance gene in VPKS1 and VPLS2. Lanes 7, 8: chloramphenicol resistance gene in VPDS4 and VPIS1. Lanes 9, 10: erythromycin resistance gene in VPCW3 and VPEW2. Lanes 11, 12: quinolone resistance protein gene in VPDW1 andVPKW1. Lanes 13, 14, 15: aminoglycosides resistance gene in VPLW1, VPDW1, and VPKW1.

The rate of seven resistance genes of *V. parahaemolyticus* from seawater, fish and all isolates were determined in this study, and the findings are depicted in Figures-2 and 3 and Table-6. Analysis of the findings revealed that only one gene (*strB*) from seawater isolates had 100% of the resistance gene followed by *tetA* with 64.28% of the resistance gene. The rest of the genes from this group had low rates of resistance genes with the least being from *aac(3)-lla*, which had 7.14% of the resistance gene. No gene from fish isolates possesses 100% of resistance gene seeing that the highest rate of resistance was found in *blaP1* with 80.64% of the resistance gene. The rest of the genes under this group had moderate to low rates of resistance genes and the gene with the lowest rate of resistance from this group was *floR* with 16.12% of resistance. Similarly, no gene from all isolates had 100% of the resistance gene as the highest rate of resistance gene was *strB*, which had 80.82% of the resistance gene. Most of the genes in this group had moderate-to-low rates of resistance, with the least rate of resistance with *aac(3)-lla* with 16.43% of resistance. There was no regular pattern in the rate of resistance from all the three groups.

**Table 6:** Antibiotic resistance gene patterns of *Vibrio* *parahaemolyticus*.

Resistance gene profile	Isolate codes	Pattern
*aac (3)-IIa*, *blaP1*, *floR*, *qnrA*, *strB*, *tetA*	VPDW1, VPKW1, VPLW1	A
*ermB*, *floR*, *qnrA*, *strB*, *tetA*	VPKW2, VPLW2	B
*blaP1*, *ermB*, *floR*, *strB*, *tetA*	VPCW3, VPEW2, VPFW4, VPHW4, VPIW1, VPJW3	C
*blaP1*, *strB*, *tetA*	VPLW3	D
*strB*, *tetA*	VPAW1, VPBW2, VPCW1, VPCW8, VPEW1, VPGW1, VPHW1, VPHW2, VPIW4, VPIW9, VPJW5, VPKW3, VPKW5	E
*blaP1*, *strB*, *tetA*	VPEW3, VPKW4	F
*blaP1*, *strB*	VPCW4, VPGW5	G
*strB*	VPAW4, VPBW4, VPCW2, VPCW6, VPCW7, VPHW10, VPHW3, VPHW6, VPIW6, VPJW1, VPJW2, VPJW4, VPJW6	H
*aac (3)-IIa*, *blaP1*, *ermB*, *floR*, *qnrA*, *strB*, *tetA*	VPDS4, VPIS1, VPKS3	I
*ermB*, *floR*, *qnrA*, *strB*, *tetA*	VPKS1, VPLS2	J
*aac (3)-IIa*, *blaP1*, *ermB*	VPCS2, VPES1	K
*blaP1*, *strB*, *tetA*	VPKS5, VPLS3	L
*blaP1*, *strB*, *tetA*	VPAS4, VPBS1, VPCS1, VPDS1, VPKS6, VPLS4	M
*aac (3)-IIa*, *strB*	VPAS1, VPBS2, VPDS2, VPLS1	N
*blaP1*, *qnrA*	VPBS3, VPDS5, VPHS3, VPJS1	O
*blaP1*	VPAS5, VPCS3, VPHS1, VPJS3	P
*blaP1*	VPAS3, VPAS6, VPCS4, VPIS2	Q

*strB*=Streptomycin resistance, *blaP1=*β-lactamase resistance, *flo*R=Chloramphenicol resistance, *tet*A=Tetracycline resistance, *erm*B=Erythromycin resistance, *qnrA*=quinolone resistance protein, *aac (3)-IIa*=Aminoglycosides resistance

### Percentages of antibiotic resistance genes of *V. parahaemolyticus*

**Figure-4:**
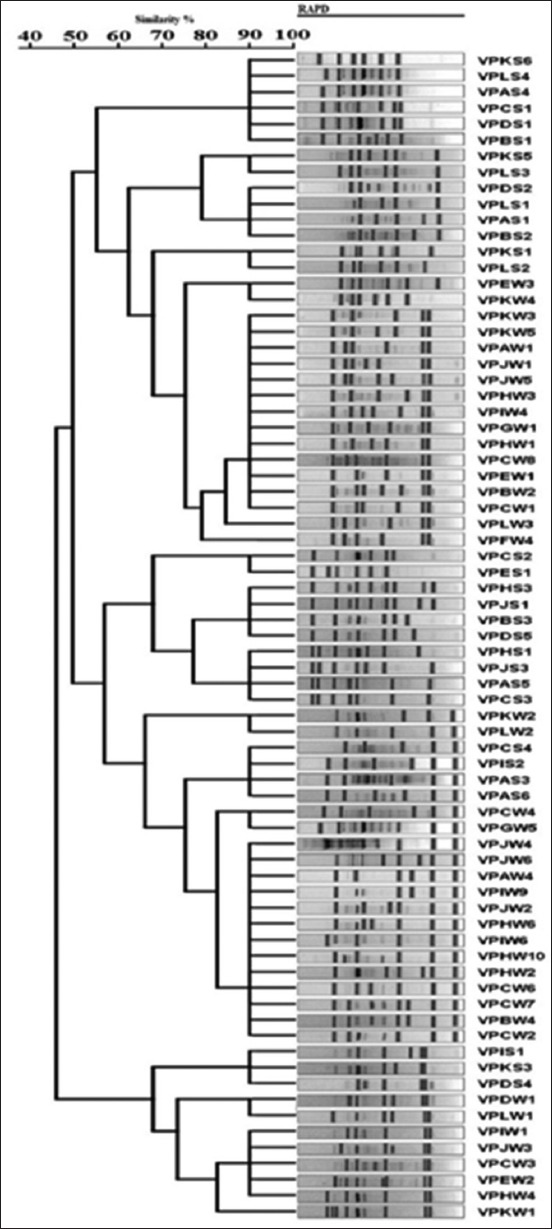
Dendrogram of typeable *Vibrio parahaemolyticus* isolates produced from randomly amplified polymorphic DNA analysis using average linkage unweighted group pair method with arithmetic averages.

Based on the analysis of the results of *V. parahaemolyticus* isolated from this study, all the isolates were multi-antibiotic-resistant as all the isolates were resistant to at least three different antibiotics, with isolates VPDW1, VPKW1, VPLW1, VPDS4, VPIS1, and VPKS3 being resistant to 13 of the different antibiotics tested (Table-3). Seventeen different antibiotic resistance patterns were observed in this study. The isolates have plasmids of varying sizes ranging from 2.7 kb to 42.4 kb even though the majority of the isolates were plasmidless. The dendrogram based on antibiotic resistance patterns of the *V. parahaemolyticus* isolates discriminated the isolates into three clusters (Figure-4).

### Molecular fingerprinting of *V. parahaemolyticus* by RAPD-PCR and ERIC-PCR typing

**Figure-5:**
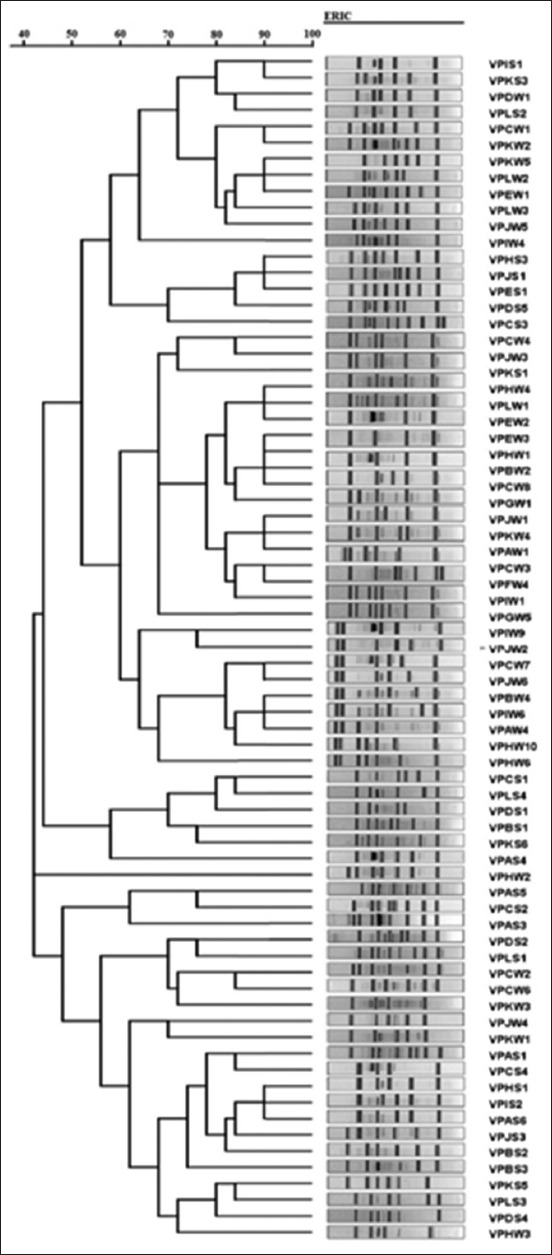
Dendrogram of typeable *Vibrio parahaemolyticus* isolates produced from enterobacterial repetitive intergenic consensus analysis using average linkage unweighted group pair method with arithmetic averages.

The dendrogram of typeable *V. parahaemolyticus* isolates produced from RAPD-PCR and ERIC-PCR analysis using average linkage UPGMA is shown in Figures-4 and 5, respectively. The RAPD-PCR analysis discriminated typeable *V. parahaemolyticus* isolate into three clusters and one single isolate. On the other hand, the ERIC-PCR analysis discriminated typeable *V. parahaemolyticus* isolates into 12 clusters and six single isolates.

## Discussion

The pathogenicity of *V. parahaemolyticus* has been reported to be largely influenced by two common virulence genes among others and these are the *tdh* and *trh* [26]. In this study, seven genes were evaluated and our analysis revealed that only one gene from seawater isolates of *V. parahaemolyticus* had a high virulence gene percentage and this was gene *tdh* which had 95.23% *tdh* gene. The gene with the least virulence percentage from seawater isolate was found to be gene *trh* (9.52%). Two genes from fish isolate have a high percentage of virulence and these were *asp* and *tdh* genes which had 83.87% and 80.64% of virulence, respectively, while two other genes (*tlh* and *tox*R) have 64.51% virulence each. These percentages of virulence genes obtained in this study are relatively high compared to those reported in an earlier study [26], where 16.2% was reported. In a related study [27], it was reported that *Vibrio* isolates possess 100% *toxR* virulence gene and this is relatively high compared to the 64.51% *toxR* reported in this study. The gene with the least virulence percentage from the fish isolate was *trh*, which had 16.12% virulence, which has been similarly reported by Tan *et al*. [26]. However, only one gene from all isolate had a high percentage of virulence and this was *tdh* with the virulence of 89.04% and one other gene (*asp*) had 60.27% of virulence. The findings of *tdh* with 89.04% of virulence gene corroborate with the findings of Oliva *et al*. [28], who reported 68.2% of virulence gene of *Vibrio* isolates to be *tdh* gene. The gene with the least virulence percent from all isolate was *trh* gene with the virulence of 12.32%. Comparatively, isolates from fish had genes with higher virulence percentage compared to seawater isolates.

Antibiotic susceptibility of *Vibrio* species has been documented in several studies [9,26]. In this study, analysis of the findings revealed that only one gene (*strB*) from seawater isolates had 100% of the resistance gene followed by *tetA* with 64.28% of the resistance gene. This was relatively very high compared to the rate reported in an earlier study [26], where *Vibrio* isolates were reported to have only 13.33% of the resistance gene. The rest of the genes from this group had low rates of resistance genes with the least being from *aac(3)-lla*, which had 7.14% of the resistance gene. These findings corroborate with those reported by Tan *et al*. [26] who reported 12.5% of *aac(3)-lla* from *Vibrio* isolated from seawater. No gene from fish isolates had 100% of resistance gene seeing that the highest rate of resistance was found in *blaP1* with 80.64% of the resistance gene. In an earlier study [26], the percentage of *blaP1* in *Vibrio* isolates from seawater was reported to be 65%, which is comparatively lower than the 80.64% found in this study. The rest of the genes under this group had moderate to low rates of resistance genes and the gene with the lowest rate of resistance from this group was *floR* with 16.12% of resistance. Similarly, no gene from all isolates had 100% of resistance gene as the highest rate of resistance gene was *strB* which had 80.82% of resistance gene which was relatively low compared to those reported by Tan *et al*. [26]. In another related study [29], the rate of *strB* resistance gene was reported to be 22.2%. Most of the genes in this group have moderate-to-low rates of resistance, with the least rate of resistance seen *aac(3)-lla* with 16.43% of resistance. There was no regular pattern in the rate of resistance from all the three groups.

Conventionally, *Vibrio* has been known to be greatly susceptible to nearly all antimicrobials. Nevertheless, recently, resistance to antimicrobials has emerged and evolved in numerous bacterial genera as a result of the excessive utilization of antimicrobial agents in human, agriculture as well as aquaculture systems [30]. In this study, all the *V. parahaemolyticus* isolated were resistant to at least three different antibiotics, with isolates VPDW1, VPKW1, VPLW1, VPDS4, VPIS1, and VPKS3 being resistant to 13 of the different antibiotics tested. This corroborates with the findings reported in other related studies [21,31], who reported variations in the pattern of antibiotic resistance of *V. parahaemolyticus*. Seventeen different antibiotic resistance patterns were observed in this study. These results of ours were inconsonant with the findings of You *et al*. [32], who reported 20 different antibiotic resistance patterns in *Vibrio* isolates. The isolates had plasmids of varying sizes ranging from 2.7 kb to 42.4 kb even though the majority of the isolates were plasmidless. You *et al*. [32] had also reported plasmid sizes ranging from 2.2 kb to 24.8 kb in *Vibrio* strains isolated in their study, as similarly found in this study. The dendrogram based on antibiotic resistance patterns of the *V. parahaemolyticus* isolates discriminated the isolates into three clusters. In an earlier related study [33], *V. parahaemolyticus* was similarly discriminated into four clusters by RAPD-PCR and ERIC-PCR analysis.

Molecular techniques for *Vibrio* identification as well as subtyping have been developed, and these include DNA microarray technologies and PCR-based techniques that target species-specific determinants. PCR-based molecular-typing studies have been performed with ERIC-PCR and RAPD analysis [34]. The RAPD-PCR analysis in this study discriminated typeable *V. parahaemolyticus* isolate into three clusters and one single isolate. These findings of ours corroborate with the findings reported in an earlier study [35], who documented that *Vibrio* isolates were discriminated into two clusters by RAPD-PCR analysis. The ERIC-PCR analysis, on the other hand, discriminated typeable *V. parahaemolyticus* isolates into 12 clusters and six single isolates. These findings corroborate with the findings of Bhowmick *et al*. [36], who reported that *V. parahaemolyticus* isolates were discriminated into 2, 4, and 10 clusters at 20%, 35%, and 45% similarity using RAPD-PCR analysis. The authors [36] similarly reported that ERIC-PCR discriminated the *V. parahaemolyticus* isolates into 3, 4, and 7 clusters at 10%, 25%, and 45% similarity. In another related study [33], *V. parahaemolyticus* was discriminated into four clusters by RAPD-PCR and ERIC-PCR analysis, which comparatively agree with the findings of this study. Yet in another study [37], *V. parahaemolyticus* was discriminated into four clusters by RAPD-PCR analysis as similarly observed in this study.

## Conclusion

In this study, *Vibrio parahaemolyticus* was isolated from four different seawaters and fish collected from Malaysian beaches. These isolates were found to have virulence genes as well as antibiotic resistance genes. They were also found to be multi-antibiotic-resistant. The isolation of *V. parahaemolyticus* species with virulence genes and antibiotic resistance genes in Malaysia beaches and fish is an interesting finding, and the information generated in this study could be used to guard the public on the possible public health risk in these recreational beaches as well as consumption of infected fish.

## Authors’ Contributions

OMF and AA designed the study protocol. OMF, AAS, KMY, MGA, and BJM were involved in sample collection. OMF wrote the paper while OMF, AAS, and AA were involved in drafting the manuscript. All authors revised, read, and approved the final manuscript.
